# Characteristics and treatment strategies of the hip fracture triad

**DOI:** 10.3389/fsurg.2025.1510344

**Published:** 2025-03-11

**Authors:** Lin Li, Lianxin Li, Dongsheng Zhou, Qin Zhao, Ci Li

**Affiliations:** ^1^ Department of Orthopedics, Shandong Provincial Hospital Affiliated to Shandong First Medical University, Jinan, Shandong, China; ^2^ Department of Rehabilitation Medicine, Shandong Provincial Hospital Affiliated to Shandong First Medical University, Jinan, Shandong, China

**Keywords:** acetabular fracture, femur fracture, dislocation, hip fracture, management

## Abstract

**Objective:**

To explore the clinical characteristics and treatment strategies of the hip fracture triad (acetabular fracture, hip dislocation combined with proximal femur fracture).

**Methods:**

A retrospective analysis was performed on 11 patients with hip fracture triad admitted to Shandong Provincial Hospital from January 2014 to December 2020. There were 9 males and 2 females; age (38.7 ± 12.2) years old (range 12–53 years). After all patients are admitted to the hospital, a treatment plan will be formulated based on the fracture type and associated injuries, and long-term follow-up will be conducted.

**Results:**

This study included clinical data of 11 patients with hip fracture triad, of which 9 cases were treated surgically and 2 cases were treated conservatively. All patients were followed up. 9 patients successfully completed the operation. The operation time was (4.4 ± 1.4) hours (range 3–8 h); intraoperative bleeding was (600.0 ± 355.9) ml (range 400–1,200 ml). Fracture reduction was evaluated according to the acetabular fracture Matta score: 7 cases were excellent, 2 was good, and none was poor; 2 patients with old injuries chose conservative treatment as the final treatment plan. Acetabular fractures at the final follow-up were evaluated using the modified Merle d'Aubigné-Postel score of the hip joint: 7 cases were excellent, 1 was good, and 3 were poor. 1 patient developed traumatic hip arthritis after surgery, underwent total hip arthroplasty, and recovered well after surgery; 1 patient underwent hemihip arthroplasty 1 year after surgery due to femoral neck fracture and recovered well after surgery; 1 patient suffered from cerebral infarction complicated by long-term bed rest, poor hip joint mobility and basic loss of self-care ability; 2 patients with conservative treatment of old fracture had limited hip joint functional mobility, unequal length of both lower limbs, and poor hip joint mobility.

**Conclusion:**

The hip fracture triad is a complex, high-energy injury that is extremely rare clinically. A correct understanding of the characteristics and mechanism of this type of injury, and prompt and effective treatment strategies, will help improve patient prognosis. Surgery is the preferred treatment option for this injury, and early reduction or lower limb traction can help reduce the occurrence of postoperative complications.

## Introduction

**Figure 1:**
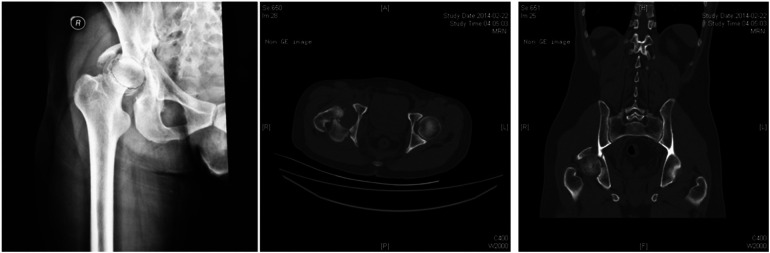
The typical case of the hip fracture triad. A 27-year-old male patient was admitted to hospital with right hip pain and limited mobility for 7 h after being injured in a car accident. He was diagnosed with right acetabular fracture, right hip dislocation, and right femoral neck fracture. Manual reduction under anesthesia was attempted in an emergency, but the reduction failed and right lower limb traction (femoral condylar traction) was used instead. Preoperative CT scan showed that acetabular fractures were classified as acetabular transverse and posterior wall fractures according to Letournel Judet classification of acetabular fractures, hip dislocation was classified as posterior hip dislocation according to the direction of femoral head dislocation, and femoral neck fractures were classified as subcephalic femoral neck fractures according to the location of the fracture line.

Floating hip injury is a severe peri-hip fracture that can cause the injured hip to float. The traumatic kinetic energy that causes a floating hip injury is enormous. The patients with floating hip injury have high disability rate, high mortality rate and complex injury condition, so there is no unified and comprehensive classification and treatment standard. At present, it is considered that there are two classifications about floating hip injury. Liebergall's basal injury in the Tile classification of the pelvis, Floating hip injuries were classified into 3 types according to whether they were combined with femoral and acetabular fractures (1). Muller classification emphasizes the integrity of the pelvic ring (2). There are published researches have emphasized the severity, difficulty of management, and a high number of complications of this injury (3, 4). However, there exist a kind of rare injury which is characterized by: acetabular fracture combined with hip dislocation and proximal femoral fracture. This type of injures has never been reported. The typical case is shown in Figure 1.

High-energy violence is often required to generate complex fracture. Due to the abundant blood supply of the pelvis or acetabulum and femur, once these structures are fractured, they are prone to massive blood loss and serious complications such as traumatic shock, retroperitoneal hematoma, urinary system injury, and abdominal organ damage, which can cause death and disability (5). Therefore, the aim of this study was to better define and characterize this injury and try to provide additional data to indicate the rule of treatment.

## Methods and patients

All the patients with hip fracture triad who were treated at Shandong Provincial Hospital from January 2014 to December 2020.

### Inclusion and exclusion criteria

Inclusion criteria: (1) Patients with acetabular fractures, hip dislocation combined with proximal femoral fractures, including femoral head, femoral neck and intertrochanteric fractures; (2) Use active treatment strategies to intervene, surgery or conservative treatment; (3) The main outcome measures were fracture reduction, hip movement and complications. (4) Retrospective serial case study. Exclusion criteria: (1) Patients who had a previous hip trauma history; (2) Incomplete clinical or imaging follow-up data; (3) The follow-up time was less than 1 year.

### Collected data

The following data for each patient were collected during the patients' hospitalization, incuded gender, age, fracture type, cause of injury, associated injuries, open fracture (yes or no), operative approach, fracture fixation, ligamentous injury (yes or no) and repair methods, follow-up time, complications. Matta score and Merle d'Aubigne ⁃Postel score (6) were performed at the last follow-up visit and used to assess the hip joint improvement.

### Patient management

The ISS score was used to evaluate the overall condition of the patient's injury. If the patients still had haemodynamic instability after initial treatment, ATLS therapy should be initiated immediately. The treatment strategy was formulated according to the Damage Control Orthopedics (DCO) principle. For such patients, active fluid resuscitation, blood transfusion if necessary, and patients' vital signs should be closely monitored. Meanwhile, simple and rapid imaging examination (pelvis, ilium oblique and obturator oblique X-ray, CT and three-dimensional reconstruction, etc.) was performed to assist in assessing the degree of injury. For patients who meet the surgical conditions, the appropriate position and surgical approach should be selected according to the type of pelvic and acetabular fracture injury, and the appropriate internal fixation should be selected for fixation. Low molecular heparin was routinely used to prevent deep venous thrombosis of lower extremities. After the operation, the normal diet was gradually restored, and rehabilitation training was carried out according to the healing of the fracture.

### Statistical analysis

The statistical analysis was conducted using SPSS version 25.0 statistical software (SPSS Inc., Chicago, Illinois, USA). Mean ± standard deviation was used for measurement data. Counting data were presented as percentages. Value of *p* below 0.05 was considered as statistically significant.

## Results

### Demographic data

During the study period, 283 patients were diagnosed pelvis fractures combined with femoral fracture in our institution, and 11 patients met the inclusion criteria, including 9 cases of fresh fracture and 2 cases of old fracture. There were 9 males and 2 females patients, in the age range 12–53 years, average 38.7 ± 12.2 years. The main cause of injuries was falling injury from height, and there were 6 patients. Five patients suffered from a traffic injury (Two patients was a motorcycle–car accident, and the others was a pedestrian–car accident). There were 7 cases of fresh fractures within 3 weeks and 4 cases of obsolete pelvis fracture. The injury severity score (ISS) range (range 9–32), average 16.7 ± 7.7 scores. Among them, 7 patients were complicated with fractures of other parts, 1 patient with craniocerebral injury, 2 patients with thoracic organ injury, 1 patient with abdominal organ injury, and 2 patients with sciatic nerve injury. The details are shown in Table 1.

**Table 1:** Demographic and the hip fracture triad information.

Variables	Data	
Demographics		
Mean age (years)	38.7 ± 12.2	
Injury severity score, ISS	16.7 ± 7.7, range 9–32	
Gender	Number	Percent
Males	9	81.8%
Females	2	18.2%
Mechanism of injury		
Road traffic accident	6	54.5%
Falling injury from height	5	45.5%
Fracture site		
Left limb	5	45.5%
Right limb	6	54.5%
Acetabular fracture		
Simple type	6	54.5%
Complex type	5	45.5%
Dislocation pattern		
Anterior dislocation of hip	0	0
Posterior dislocation of hip	8	72.7%
Central dislocation of hip joint	3	27.3%
Fracture of proximal femur		
Fracture of femoral head	5	45.5%
Fracture of femoral neck	4	36.3%
Intertrochanteric fracture of femur	2	18.2%

### Therapeutic measures

All patients were closely monitored and evaluated. After the patient's condition is stabilized, the appropriate surgical approach and internal fixation method are selected according to the actual situation. During the procedure, we first immobilize the femur and then the pelvic or acetabular fracture. The mean time from injury to femoral fixation was 6.1 days. Operation time (4.4 ± 1.4) h (range 3–8 h); Intraoperative bleeding (600.0 ± 355.9) ml (range 400–1,200 ml; Table 2).

**Table 2:** Operative data and outcomes.

Variables	Numbers	Percent
Injury to surgery time (days)	6.3 ± 2.4	-
Hospital stays (days)	18.7 ± 4.6	-
Operation time (h)	4.4 ± 1.4 (range 3–8 h)	-
Amount of bleeding (ml)	600.0 ± 355.9 ml (range 400–1,200 ml)	-
Definitive femoral fixation		
Nail (%)	8	88.9%
Locking plate (%)	1	11.1%
Definitive acetabular fixation		
Anterior approach (%)	2	18.2%
Posterior approach (%)	3	27.3%
Combined approaches (%)	4	36.3%
Total hip arthroplasty (%)	0	0
Non-surgical treatment (%)	2	18.2%
Definitive pelvic ring fixation		
Anterior ring only	2	22.2%
Posterior ring only	6	66.7%
Anterior and posterior rings	1	11.1%
Quality of reduction		
Matta score		
Excellent	7	77.8%
Good	2	18.2%
Poor	0	0
Merle d'Aubigné-Postel		
Excellent	7	63.6%
Good	1	9%
Poor	3	27.3%

### Radiological and clinical outcomes

According to the Matta's criteria, anatomical reduction was achieved in 9 patients. The overall excellent and good rate reached 81.8%. Merle d'Aubigne ⁃Postel score was used at the last follow-up, of which 7 cases were excellent, 1 case was good and 3 cases were poor. Among the 9 patients who underwent surgical treatment, 8 patients could walk normally, and 1 patient basically lost the ability of self-care due to severe injury and long-term bed rest complicated with cerebral infarction. There were 2 patients with hip fracture triad who had been treated conservatively for more than 3 months, and finally chose conservative treatment due to serious injury, fracture malunion, and economic reasons.

### Complications

In this group of 9 patients, all the incisions healed in the first stage after surgery, and none of them had complications such as aggravated nerve injury, pressure sore, loosening or breakage of internal fixation, and iatrogenic nerve and vascular injury. One patient suffered from posttraumatic osteoarthritis after operation and underwent total hip arthroplasty. One patient underwent artificial femoral head replacement due to femoral neck fracture 1 year after operation and recovered well. The follow-up of 2 patients with old fracture showed that the functional activity of the hip was limited, the length of both lower limbs was severely unequal, and the hip activity was poor.

## Discussion

The unique pestle-mortar structure of the hip joint is the anatomical basis for its good stability (7). Severe acetabular and proximal femur fracture usually result from high-energy trauma and are combined with head, chest, abdomen or other injuries (8, 9). Such injuries are relatively rare. Although there is broad consensus on the management of unstable acetabular fractures, hip dislocation and femoral fractures, few reports have discussed the treatment protocols and outcomes of patients with hip fracture triad. There are few relevant studies on this injury, even case reports.

### It is different from floating hip injury

The floating hip injury refers to a fracture of the pelvic ring or acetabulum and an ipsilateral femoral fracture (5). By definition, hip fracture triad is similar to floating hip injury, but it is not identical, with the main difference being whether the hip is dislocated. Hip dislocation is usually caused by high-energy trauma (10). In hip fracture triad patients, the dislocation of the joint capsule, associated blood vessels and round ligament were severely lacerated, and the nutrient vessels of the femoral head are also damaged. Meanwhile, the length of time between dislocation and reduction is related to the degree of ischemic changes, chondrolysis, and degeneration of the femoral head (11). The incidence of aseptic necrosis of the femoral head within 6 h of reduction was 5% Over 6 h reset can be up to 50% (12). In our case, there was no avascular necrosis of the femoral head. Although it was difficult to complete the hip reduction within 6 h, we completed the lower limb traction as soon as possible, which may benefit the self-repair of the muscles and joint capsule, as well as the restoration of blood circulation in the femoral head. At the same time, the location of the proximal femur fracture may also be the reason for not causing the necrosis of the femoral head. Posttraumatic osteoarthritis is one of the most common long-term complications of hip dislocation (13). In one case, hip posttraumatic osteoarthritis occurred and total hip replacement was completed, and the symptoms of osteoarthritis were significantly relieved after surgery.

### Injury mechanism and fracture patterns

The mechanism of injury in the hip fracture triad is complex. Such injuries may be caused by great violence from the greater trochanter or lower limb to the femoral head, where the femoral head impinges on the acetabulum causing fractures of the proximal femur and acetabulum, while rotating the proximal femur causes dislocation of the hip (14).

The triad of hip fracture is mainly divided into acetabular fracture, hip dislocation and proximal femoral fracture. At present, there are relatively complete and mature types of injuries in each part. According to Letournel-Judet classification (6), acetabular fractures can be divided into complex fractures and simple fractures. According to the anterior column of the acetabulum, anterior wall of the acetabulum, posterior column of the acetabulum, the posterior wall of the acetabulum are divided into 10 fracture types. Hip dislocation can be divided into posterior hip dislocation, central hip dislocation and anterior hip dislocation according to the direction of the femoral head. Proximal femoral fractures can be divided into femoral head fractures, femoral neck fractures and intertrochanteric fractures. Among the 11 patients in this study, 6 (54.5%) of the acetabular fractures were simple posterior wall fractures and 5 (45.5%) were complex acetabular fractures. Posterior dislocation (8 cases, 72.7%) and central dislocation (only 3 cases, 27.3%) were most common. At present, no patients with “hip fracture triad” combined with anterior dislocation of the hip have been found, and its cause needs further study. Among the proximal femoral fractures, there were 5 cases of femoral head fracture (45.5%), 4 cases of femoral neck fracture (36.3%), 1 case of intertrochanteric fracture (9.1%), and 1 case of femoral neck fracture combined with greater trochanteric fracture (9.1%).

### Preoperative preparation and surgical timing of hip fracture triad

The triad of hip fracture is a kind of high-energy injury, which is often combined with serious combined injury, and can seriously affect the function of the patient's hip joint and cause serious consequences of lifelong disability. Early active and effective preoperative intervention and reasonable choice of operation time can affect the prognosis of patients. After admission, patients were treated under the guidance of damage control orthopaedics (DCO) principles, actively managing life-threatening injuries and managing hip dislocation in the early stages when the condition was relatively stable (15).

For hip dislocation, manual reduction can be attempted in the early stage to restore a good head-mortar matching relationship, which can preserve the blood supply of the femoral head and reduce the occurrence of complications such as traumatic arthritis and avascular necrosis of the femoral head in the later stage. The study found that early hip reduction is the most important factor affecting the patients with advanced femoral head necrosis (16). However, in patients with hip fracture triad, both the upper and lower ends of the hip joint are fractured, and it is difficult to find a good fulcrum in the process of manual reduction, and forced reduction is not only difficult to succeed, but also may cause the risk of re-fracture and fracture. Therefore, for the failure of reduction of posterior hip dislocation and central hip dislocation, early lower limb traction can be maintained until preoperative, which can not only effectively relieve the pain of patients, but also win a good opportunity for the next surgical treatment. All the 9 patients received traction before operation, and the pain of the patients was effectively relieved after traction.

The timing of operation is very important to the prognosis of patients. All the 4 cases of old acetabular fractures were transferred to other hospitals, and two of them had been admitted for more than 3 months, and no effective surgical treatment was performed in the early stage, and the pelvic fractures had malunion. Complications such as limited hip mobility, unequal length of lower limbs, and pain were found in the current follow-up. Among the 2 old patients treated by surgery, one of them received active surgical treatment 42 days after injury and artificial femoral head replacement 1 year later. All the 7 patients with fresh fracture underwent operation within 2 weeks, and all of them achieved satisfactory results with no obvious complications and most of them could walk normally.

### Surgical sequence

The sequence of fracture fixation is conducive to better reduction (17). Our treatment experience follows the principle of “first simple, then complex”. The first thing that needs to be solved is the reduction and fixation of the proximal femur fracture, because the stabilization of the femur fracture can facilitate further traction, reduction and fixation of the acetabular fracture and hip dislocation. This is also consistent with the way some concepts in the treatment of floating hip injuries (3, 18). In addition, the operation time and operation sequence should follow the DCO principle to ensure the stability of the physiological state of the patient during the operation (19, 20).

### Surgical technique

**Figure 2:**
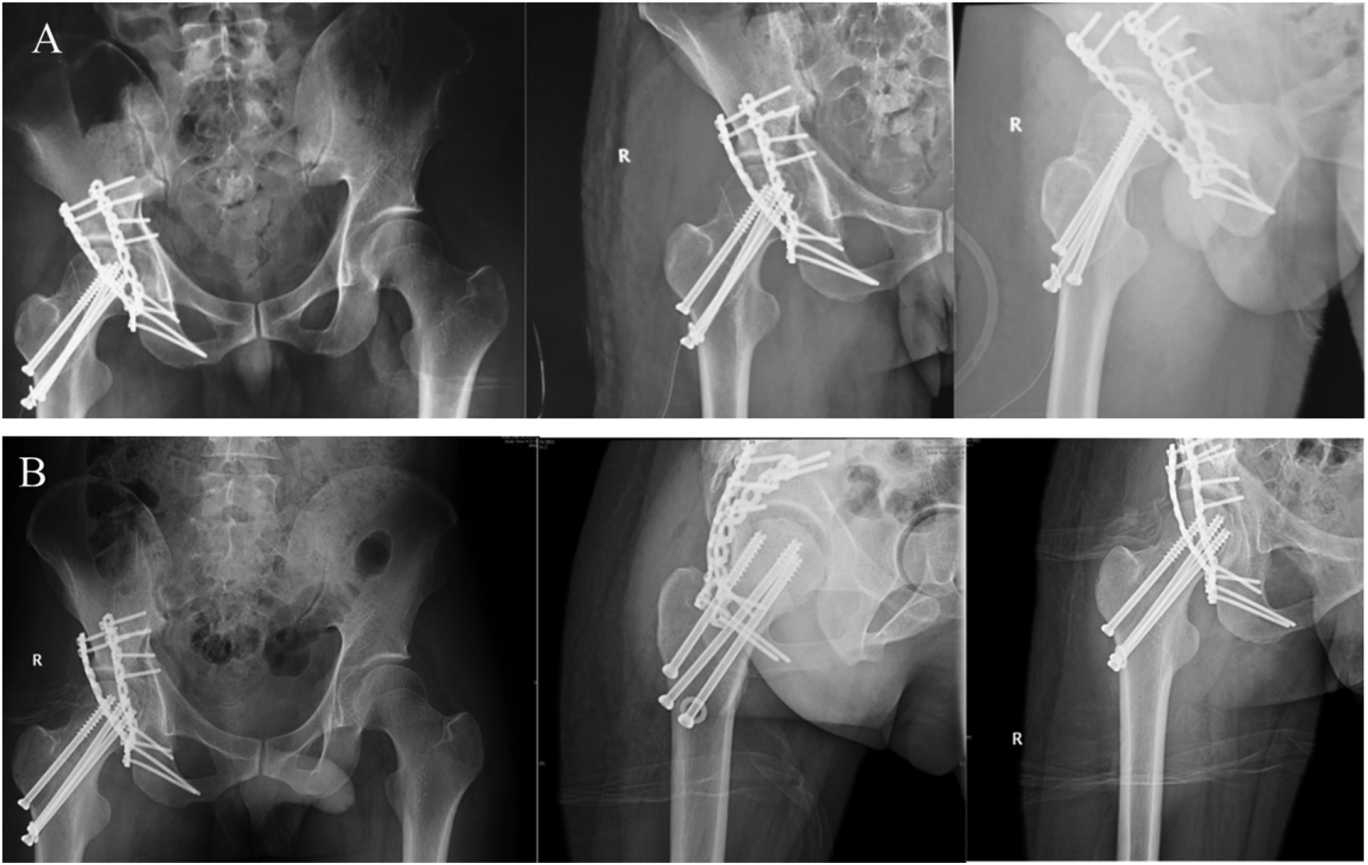
The postoperative review and 1 month follow-up of hip fracture triad. **(A)** The patient was placed under general anesthesia in the left lateral position. The K-L approach of the right hip was taken to separate and expose the femoral neck fractures, posterior hip dislocation and posterior wall fractures of the acetabulum layer by layer. The femoral neck fracture was first reduced and the hip joint was fixed with three hollow screws. The posterior wall and posterior column fractures of the acetabulum were fixed with two reconstruction plates. **(B)** Radiographs of pelvis, ilium obliquity and obliquity of obliquity 1 month after operation showed the general structure of hip joint.

For patients with combined femoral head fracture, the Kocher-Langenbeck (K-L) approach can be used to expose the load-bearing part of the femoral head fracture and the anterior part of the femoral head fracture (21). After reduction, the femoral head fracture was fixed with screws, while K-L approach could simultaneously expose the posterior wall fracture of the acetabulum. For patients with combined femoral neck and intertrochanteric fractures, the fracture can be reduced and fixed under direct vision through the rear K-L approach, and a small anterior incision can be combined if necessary. Hip dislocation can be significantly improved by manual reduction and lower limb traction. Part of the bone is embedded in the hip joint, and the hip joint can be exposed by surgical incision under direct view, and a good matching relationship can be restored under assistant traction. The acetabular injury of hip fracture triad is complicated, and the surgical approach should be determined according to the specific type of acetabular fracture. Simple acetabular fractures can be reduced by a single approach (K-L approach, ilioinguinal approach, etc.), while complex acetabular fractures should be fully considered in terms of fracture shape, and a combined anterior and posterior surgical approach can be used if necessary (22). Some scholars suggests that conservative treatment is also feasible for fractures of the posterior wall of the acetabulum without significant displacement (8). Among the patients with early surgical treatment in this study, 5 patients with simple fracture were successfully completed by a single approach, while only 1 of 4 patients with complex acetabular fracture were successfully completed by a combined anteropodial-posterior surgical approach, and all patients obtained anatomic reduction or satisfactory reduction. In our experience, the outcomes of patients which suffer from acetabular fracture combined with ipilateral femoral head fracture is poor. If the fracture area of the posterior wall of the acetabular is greater than 40% or the displacement is greater than 2 mm, and the fracture area of the femoral head is greater than 20%, the application of surgical open reduction and internal fixation can improve the prognosis. The postoperative review and 1 month follow-up are shown in Figure 2.

The patients with acetabular fracture combined with ipilateral femoral neck fracture often had a poor prognosis due to serious complications such as femoral head necrosis, among which the incidence of femoral head necrosis increased significantly when combined with hip dislocation (23). However, there is still some controversy as to whether such patients should receive semi-hip replacement in one stage. In this study, one patient underwent semi-hip replacement due to femoral head necrosis. Although surgery can effectively improve the prognosis, Pascarella et al. (24) believe that the occurrence of postoperative complications is significantly related to high-energy injury immediately generated by trauma.

This study also has the following shortcomings. In addition, this study is a retrospective study with a small sample size, and the resulting results may have certain biases. In the future, we will further expand the collection of clinical cases and conduct prospective studies. At the same time, it is not considered that the experience of the surgeon may have some influence on the results of the study.

## Conclusion

To sum up, hip fracture triad is a relatively rare serious high-energy injury, which is more common in young and middle-aged men, often combined with serious combined injury, and is relatively rare clinically. The surgeons should strengthen the understanding of the mechanism of this kind of injury, so as to formulate reasonable and effective treatment strategies to reduce the occurrence of complications. Early surgical treatment is the preferred treatment for this type of injury, which is essential to improve patient survival. Early reduction or lower limb traction can help reduce postoperative complications.

## Data Availability

The original contributions presented in the study are included in the article/Supplementary Material, further inquiries can be directed to the corresponding author.
